# Highly responsive and selective ozone sensor based on Ga doped ZnS–ZnO composite sprayed films

**DOI:** 10.1039/d3ra06959a

**Published:** 2024-01-02

**Authors:** T. Laribi, R. Souissi, S. Bernardini, M. Bendahan, N. Bouguila, S. Alaya

**Affiliations:** a Université de Gabès, Laboratoire de Physique des Matériaux et des Nanomatériaux appliqué à l'environnement, Faculté des Sciences de Gabès Cité Erriadh, Zrig 6072 Gabès Tunisia; b Carthage University, Laboratoire des Matériaux, Molécules et Applications IPEST BP51 La Marsa 2070 Tunis Tunisia riadhsouissi1@gmail.com; c Aix Marseille Univ, CNRS, IM2NP Marseille France

## Abstract

Ozone detection is currently the subject of wide scientific and technological research, motivated by its harmful impact on human safety, environment and health. With the aim of searching for new highly sensitive materials for ozone detection, Ga-doped ZnS and ZnS–ZnO films were deposited by a spray pyrolysis technique. The obtained films were annealed at 400 °C for two hours. The ozone sensing properties were investigated by measuring the sensor resistance for several ozone concentrations ranging from 30 to 120 ppb. The sensor response reveals a dependence on the gallium concentration. The best response was obtained with 4% doping gallium. The sensitivity is 4.5 ppb^−1^ at 260 °C and the response to 30 ppb ozone is 150. Moreover, the sensor shows high performance such as good selectivity and fast rapidity.

## Abstract 2

Ozone detection is currently the subject of wide scientific and technological research, motivated by its harmful impact on human safety, environment and health.

## Graphical abstract

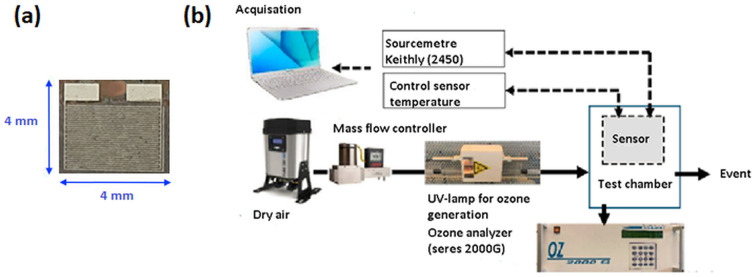

## 1 Introduction

In recent years, zinc sulfide (ZnS) and zinc oxide (ZnO) have garnered significant interest as highly promising II–VI semiconductor compounds with excellent characteristics. They exhibit a range of properties that contribute to their potential for fabricating photonic, optical, and electronic devices such as solar cells,^1,2^ window layers^3,4^ and photocatalysis.^5^ With a wide bandgap ranging from 3.54 to 3.91 eV and from 3.37 to 3.72 eV for ZnS and ZnO respectively, heterostructures of these compounds are candidates for ultraviolet lasers and detectors.^6,7^ In the literature, ZnS/ZnO compounds have been reported to be fabricated by different methods, such as: ZnS/ZnO heterostructures synthesized *via* a simple thermal evaporation method,^6,8^ ZnS–ZnO nanocomposites through the hydrothermal route,^9^ coaxial nanowires and hierarchical nanowires by chemical bath deposition and chemical etching processes, respectively,^10^ and ZnS/ZnO films *via* pulsed laser deposition.^11^ In another study,^12^ ZnS/ZnO:Mn layers were prepared by thermal sulfidation of ZnO:Mn films deposited on a Si substrate with an RF magnetron sputtering technique. In addition, Deepa *et al.*^13^ have deposited zinc sulfide thin films by ultrasonic spray pyrolysis. Obtained films have traces of the ZnO phase together with the dominant ZnS phase. Among these methods, spray pyrolysis is an interesting deposition technique for preparing ZnS/ZnO thin films. Indeed, it is a simple and an inexpensive technique to obtain optically smooth, uniform and homogeneous layers.

Recently, researchers have shown that ZnS/ZnO could be a good candidate for gas detection. Tsai *et al.*^14^ reported that ZnO/ZnS core–shell structures hydrothermally grown on SiO_2_ substrates show promise for future hydrogen sensing applications. More recently,^15^ they demonstrated that CO gas sensing measurements revealed that incorporation of a ZnS shell on ZnO nanorods can increase gas sensing capability. In another work, Ding *et al.*^16^ reported that ZnO nanowires were modified with an optimal amount of ZnS to form nanostructured heterojunctions for high-performance H2S gas sensing. Furthermore, Park *et al.*^17^ reported that ZnS-core/ZnO-shell nanowires demonstrate substantial improvement in the response to NO_2_ gas by UV irradiation. As ozone (O_3_) is a derivative gas from NO_2_, to the best of our knowledge, there is no report on the characterization of ZnS/ZnO in ozone sensing application. This prompts us to investigate ZnS/ZnO within the framework of this goal in this report. Ozone plays a vital role in supporting life as it acts as a primary UV protective layer in the stratosphere. It is a significant environmental pollutant, known to cause detrimental effects on human health even at low concentrations. Exposure to tropospheric ozone can lead to irritation of the eyes, nose and throat, as well as respiratory issues such as coughing and headaches.^18,19^ To ensure the well-being of the public, the World Health Organization (WHO) has established guidelines limiting exposure to ozone at a maximum average concentration of 100 μg m^−3^ (50 parts per billion, ppb) for 8 hours period.^20^ Additionally, for the protection of vegetation, the European directive employs an accumulated ozone over the threshold of 80 μg m^−3^ (40 ppb, designed as AOT40). Several materials have already been studied for ozone detection such as Co_3_O_4_,^21^ ZnO/SnO_2_,^22^ In_2_O_3_,^23^ WO_3_ (ref. 24) and CuAlO_2_.^25^ Generally, the selectivity of the sensors can be adjusted over a wide range by varying several aspects such as the crystal structure and morphology, the dopants used,^26^ the contact geometries,^27^ the operating temperature or the operation mode.^24^

In this paper, we demonstrate that ZnS–ZnO material is a good ozone sensor. Gallium doping improves its performances at high temperature (260 °C) and leads also to ozone detection at lower temperatures down to 120 °C. The sensing properties of the prepared films were studied using DC electrical characterization.

## 2 Experimental

ZnS–ZnO films were grown on Si/SiO_2_ substrates using the spray pyrolysis technique. This method is a simple and cost effective-way to deposit large area thin films at moderate temperatures (100–500 °C). The films were prepared by spraying an aqueous solution onto the substrates. The solution contained zinc chloride (ZnCl_2_) and thiourea [SC(NH_2_)_2_], as sources of Zn^2+^ and S^2−^ ions, respectively, with an initial Zn:S molar ratio of 1. Distilled water was used as a solvent. To achieve various levels of gallium (Ga) doping, gallium sulphate Ga_2_(SO_4_)_3_ with three different concentrations (0%, 2% and 4% atomic concentration) was added to the starting solution. The substrates were placed on a hotplate and heated gradually from ambient temperature until reaching the desired deposition temperature of 350 °C. This gradual heating helps prevent thermal shock. The spray duration was kept at 3 minutes. The distance between the spray nozzle and the substrate was fixed at 25 cm to ensure proper coverage of the substrate surfaces. During the deposition, the solution and nitrogen carrier gas flow rates were maintained at 2 ml min^−1^ and 10 l min^−1^, respectively. The formation of ZnS is given by the following equation:

1 ZnCl_2_ + SC(NH_2_)_2_ + 2H_2_O → ZnS + 2NH_4_Cl + CO_2_

**Fig. 1:**
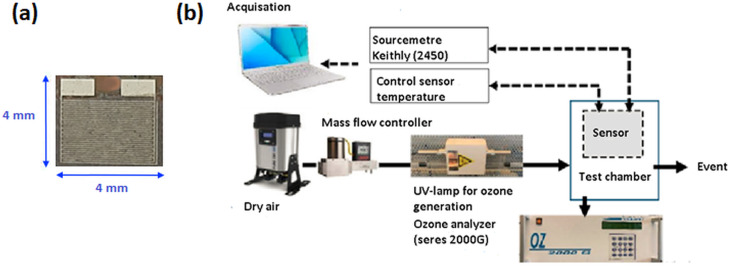
(a) Photo of platform for transducers and (b) ozone detection experimental set-up.

After deposition, thin films were annealed at 400 °C in an electric furnace under atmospheric air. The purpose of this annealing was to improve crystallization and stability of the sensitive films. The heating rate was fixed at 10 °C min^−1^ to reach 400 °C, which was maintained for 2 h. Then, the cooling was carried out in atmospheric air within the furnace. To measure the electrical resistance of the sensors, transducer platforms were used. These platforms had two interdigitated Pt/Ti electrodes sputtered on a Si/SiO_2_ substrate, as shown in Fig. 1a. The interdigitated electrodes consisted of thirty rectangular platinum fingers on a 4 × 4 mm^2^ substrate area, with 50 μm gap and finger width.

The microstructure of the deposited films was analyzed by X-ray diffraction, using a Bruker D8 Advance diffractometer, with a monochromatic Cu-Kα radiation (*λ* = 1.5406 Å). The angle 2*θ* was varied from 20° to 70° during the measurements. The morphological characterization was performed by scanning electron microscopy (SEM), using a Zeiss FE-SEM ULTRA plus microscope with an attached electron dispersive spectrometer (EDS) for chemical composition analysis.

The ozone sensing properties of the samples were investigated using the experimental set-up shown in Fig. 1b. To produce ozone gas, a UV pen ray lamp (UVP/185 nm stable ozone generator) was used. Dry air was led through a quartz tube. When illuminated by a UV lamp, some of the oxygen molecules were transformed into ozone. The UV illumination was modified by moving the shutter around the lamp. Several ozone concentrations in the range of 30 ppb to 120 ppb were obtained while maintaining the dry air flow at a fixed rate of 0.5 l min^−1^. Calibration of ozone concentrations was ensured by an Seres 2000G ozone analyzer. The sensors were exposed to ozone for 3 minutes. The operating temperature was regulated by a heat source from 120 to 260 °C. Indeed, the sensor is brought to working temperature using a resistance covered with ceramic acting as a sample holder and controlled by a stabilized generator. The heating is adjusted manually and the heating rate is practically 10° min^−1^. A Pt100 probe, fixed in the vicinity of the sample and connected to an ohmmeter, is used to display the sensor temperature. The sensors were polarized at 1 V. The resistance measurements were performed using a Keithley 2450 source-meter.

In order to calculate the response of the ZnS sensors, based on the change in resistance, we applied eqn (2) and (3) for oxidant and reducing gases, respectively:2
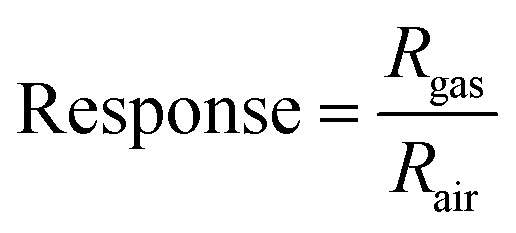
3
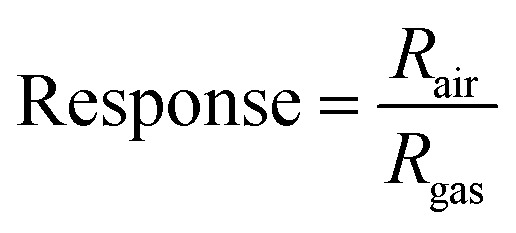
where *R*_air_ over *R*_gas_ is the ratio of the sensor resistance value in dry air and target gas, respectively.

Sensitivity is defined by the following formula:4
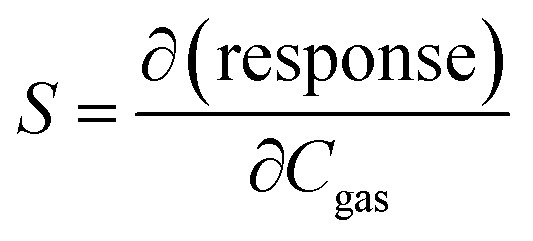
where *C*_gas_ is the concentration of the target gas. The slope of the fitting line between the response and the concentration corresponds to the sensitivity *S* of the tested sensor.

## 3 Results and discussion

### 3.1. Physical characterization

#### 3.1.1. Structural analysis

**Fig. 2:**
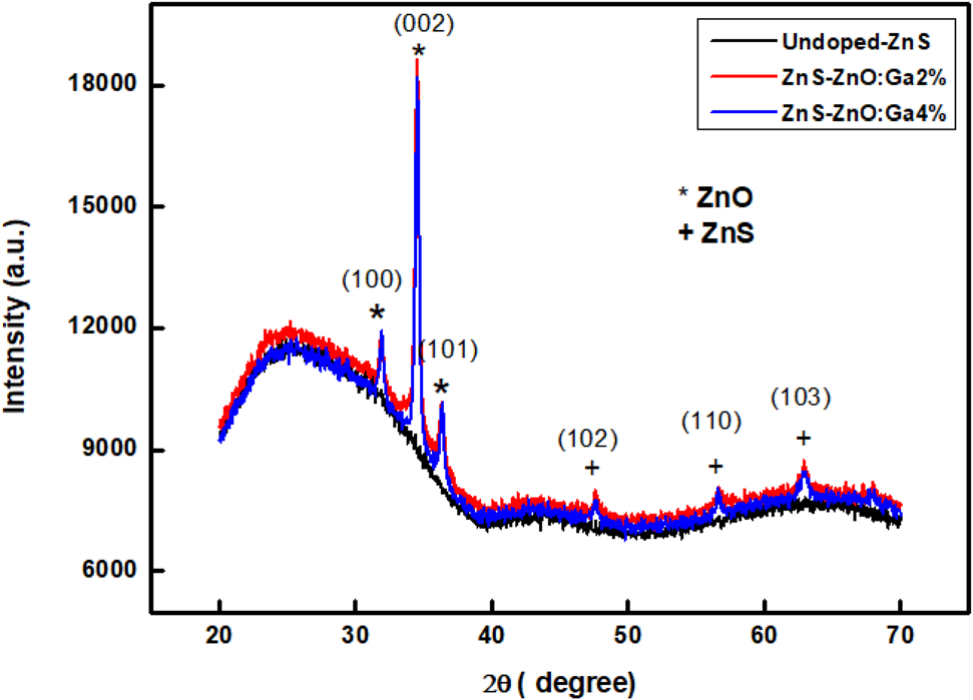
X-ray diffraction patterns thin films for different Ga concentrations.

The XRD patterns of the prepared samples are shown in Fig. 2. No characteristic XRD peak is observed for the undoped sample, revealing its amorphous structure, while those of the Ga doped exhibit narrower diffraction peaks, indicating an improved crystallinity. The observed diffraction pattern corresponds to the characteristic lattice planes of two phases: ZnS and ZnO. The three prominent peaks at Bragg angles 31°, 34° and 36° correspond to the hexagonal structure of ZnO (JCPDS card no. 36-1451) and are labeled as (100), (002) and (101). The other peaks, labeled as (102), (110), and (103) matching angles 47°, 57° and 63°, respectively, are attributed to the hexagonal ZnS phase (according to the JCPDS card no. 036-1450). Ga ions were incorporated into the hexagonal structure lattice. This indicates that the gallium doping improves the crystal structure of the films, resulting in an increase in the diffraction peak intensity. As consequence, these findings suggest that the samples have polycrystalline character and can be identified as ZnO–ZnS composite, which is in agreement with the work of Ali *et al.*^9^

Structural parameters such as crystallite size *D*, dislocation density *δ* and microstrain *ε* were estimated from the XRD profiles for the preferred orientation (002).

Assuming spherical crystallites, their size can be estimated using Debye–Scherrer's formula as follows:^26^5
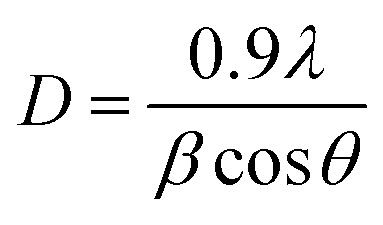
where *λ* is the X-ray wavelength (*λ* = 1.5406 Å), *θ* is the Bragg angle and *β* is the full width at half maximum (FWHM).

The dislocation density was calculated using the formula:6
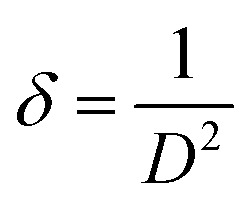


The microstrain was deduced from the following relation:7
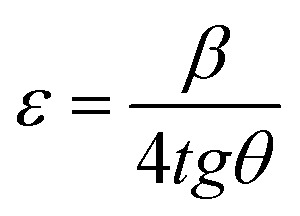


The calculated values of *D*, *δ* and *ε* are summarized in Table 1. The crystallite size is approximately 18 nm. The increase in micro-stress is attributed to the doping.

**Table 1:** Structural parameters of Ga doped ZnS–ZnO thin films

Sample	*D* (nm)	*δ* (10^10^ lines per cm^2^)	*ε*
ZnS–ZnO:Ga2%	17	35	0.007
ZnS–ZnO:Ga4%	19	26	0.062

#### 3.1.2. Chemical composition

**Fig. 3:**
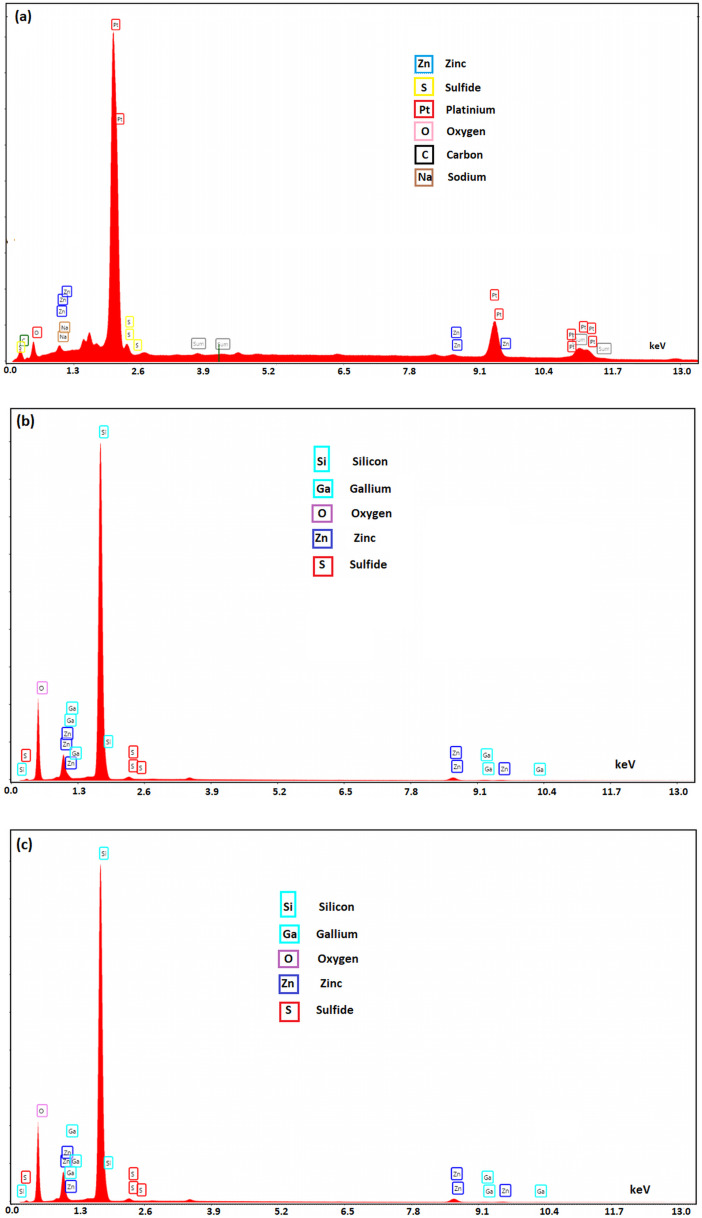
EDS spectra of (a) undoped, (b) Ga2% doped ZnS–ZnO, (c) Ga4% doped ZnS–ZnO thin films.

Quantitative elemental chemical composition of undoped and gallium-doped ZnS–ZnO was determined using energy dispersive spectroscopy (EDS). EDS spectra confirm the presence of Zn and S and showed the existence of the gallium dopant in the layer (Fig. 3). Additionally, the spectra revealed the presence of Pt, Si, and O, which are the constituents of the interdigital electrodes and the substrate.

#### 3.1.3. Morphological characterization

**Fig. 4:**
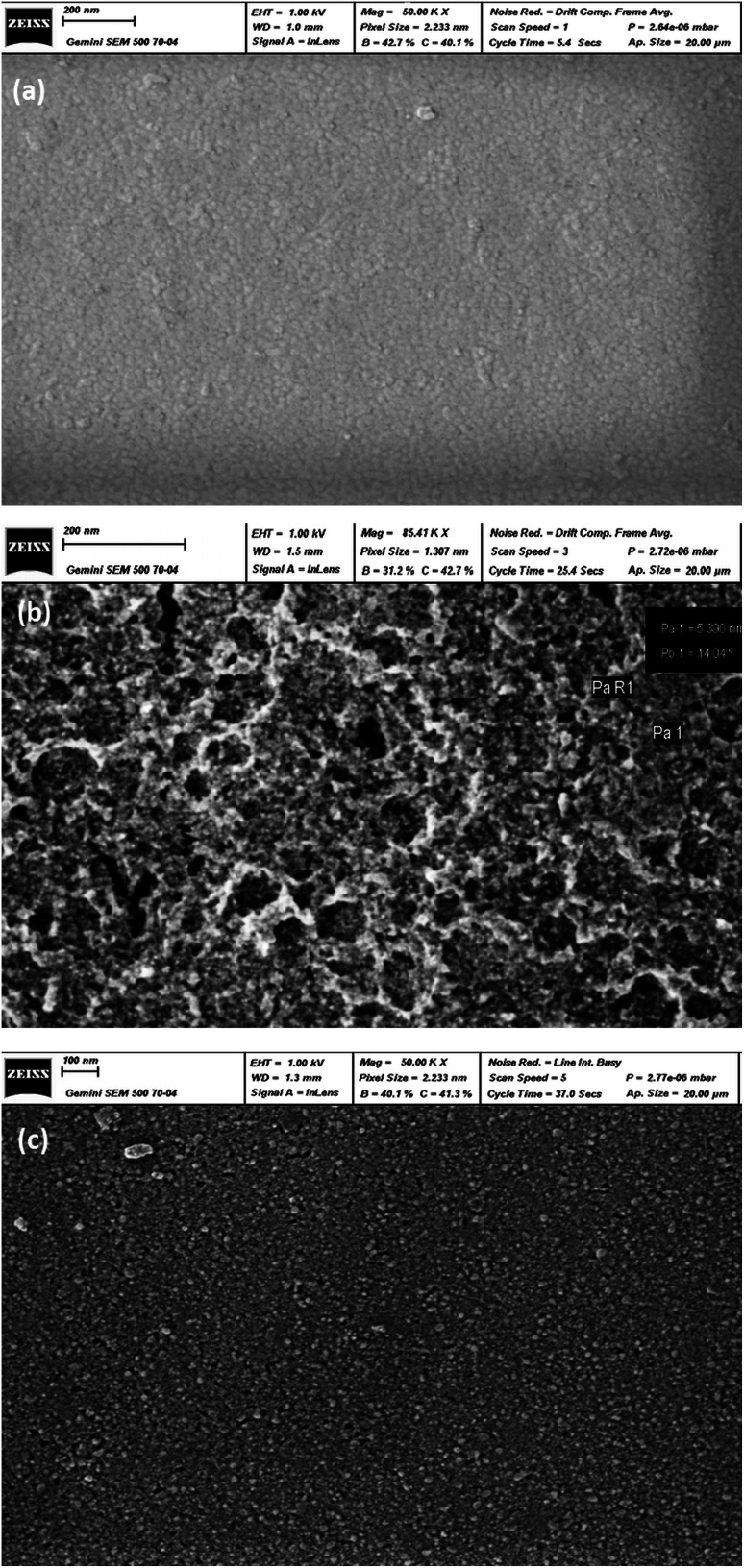
SEM graphs of (a) undoped, (b) ZnS–ZnO:Ga2% and (c) ZnS–ZnO:Ga4% thin films.

Fig. 4 shows the topographic images of all the samples, revealing a well-covered surface layer. The undoped ZnS sample (Fig. 4a) exhibits a compact layer with a smooth surface. Additionally, the grains are very small, indicating the amorphous structure of the film, which is in agreement with the XRD results. On the other hand, the image of the 2% Ga doped sample (Fig. 4b) shows the most porous layer, while the 4% Ga-doped sample (Fig. 4c) appears more compact and more granular. These results may be the consequence of a deviation from the stoichiometric composition in the starting solution. This deviation results in an incomplete reaction between the precursors, which has a very strong influence on microstructure and surface morphology. Similar results have been previously observed on In_2_S_3_ and CuInS_2_ films.^27,28^

#### 3.1.4. Optical characterization

**Fig. 5:**
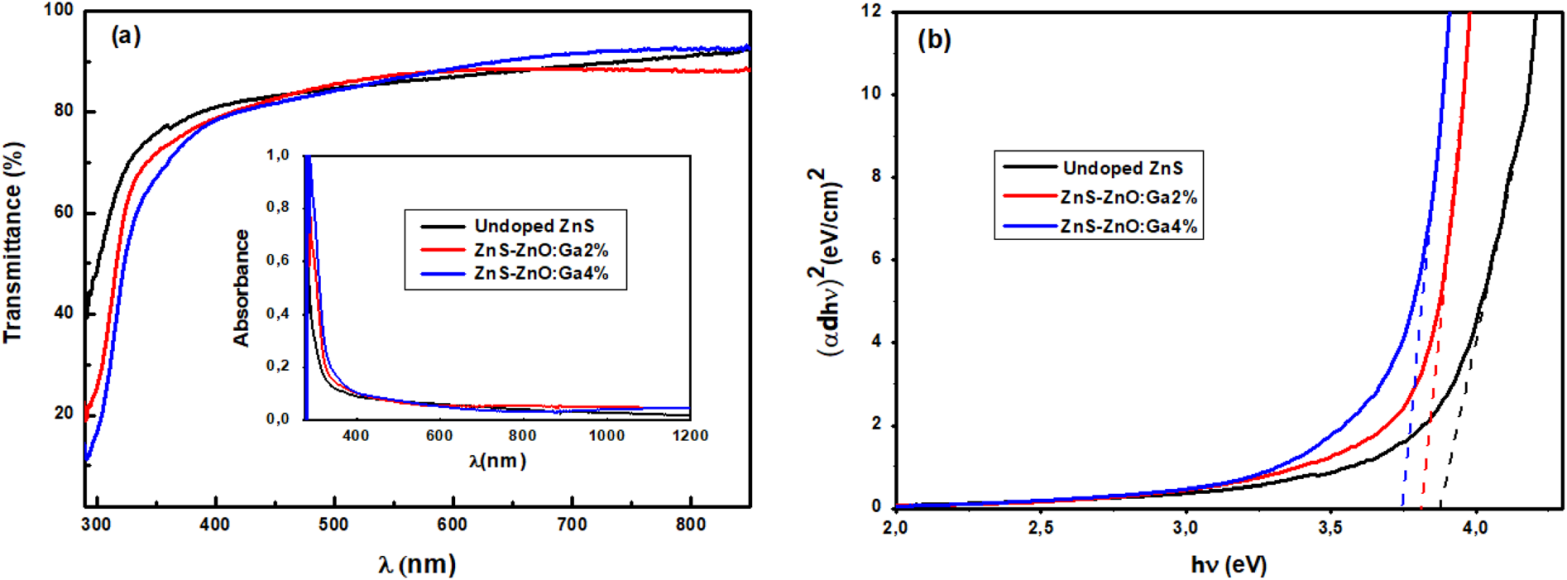
(a) Optical transmittance spectra, inset absorbance spectra, (b) Tauc plots of samples.

The transmittance spectra (T) of the different films are shown in Fig. 5a. It is clear that all the samples have high transmittance in the visible range, ranging from 80 to 90%. The effect of doping is remarkable on the absorption front. The later moves towards the long wavelengths, which makes it possible to predict a decrease in the bandgap energy with doping. This result is in good agreement with those reported by Shu-wen *et al.*,^29^ who used Na-doped ZnS, and Derbali *et al.* who used Ni-doped ZnS.^30^ Therefore, it is possible to replace CdS in CIGS solar cells by these films.^31^ In all transmittance spectra, there is an absence of interference fringes due to weak multiple reflections at the interface. In addition, the absorbance spectra in the inset of Fig. 5a show that the absorbance is high for all films in the UV-region (*λ* < 350 nm), corresponding to the fundamental absorption. Moreover, at wavelengths greater than 350 nm (Vis-IR regions), which correspond to the transparence region, the absorbance has a low value.

The absorption spectra were used to calculate the optical band gap energies of the films using Tauc expression:^32^8
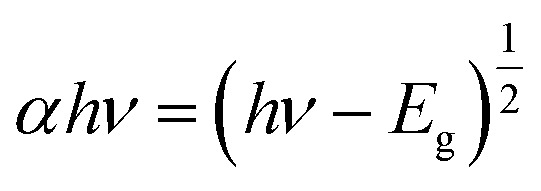
where *A* is constant and *E*_g_ is the band gap energy.

We have studied the evolution of (*αhν*)^2^ as a function of the photon energy *hν* for the different layers (Fig. 5b). The extrapolation of the linear zone of the obtained curve to the horizontal axis (*αhν* = 0) gives us a good estimate of the bandgap energy. The evaluated *E*_g_ of undoped sample is equal to 3.84 eV, which decreases slightly with doping to 3.74 eV. The bandgap energy of the undoped sample is larger than that reported for bulk ZnS. This is due to residual stress.^33^ This result is consistent with previous studies by Poornaprakash *et al.* (3.93–3.70 eV)^34^ and Wei *et al.* (3.87–3.76 eV).^35^

### 3.2. Ozone sensing study

#### 3.2.1. Ozone test and sensing mechanism

**Fig. 6:**
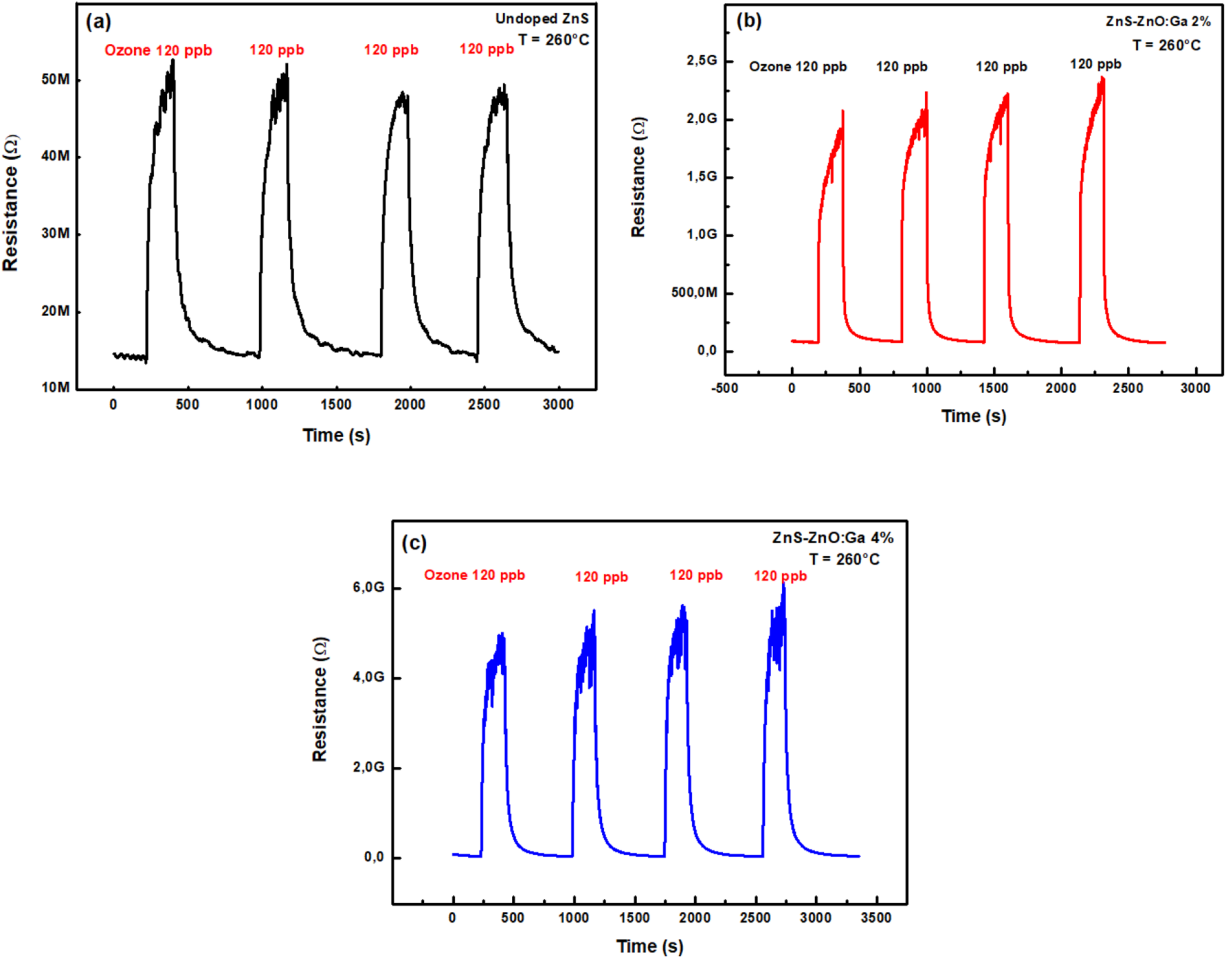
Dynamic change of electrical resistance during repetitive cycles of alternating exposure to 120 ppb ozone and dry air at 260 °C: (a) undoped, (b) ZnS–ZnO:Ga2% and (c) ZnS–ZnO:Ga4% thin films.

In order to test the sensitivity of the prepared samples to ozone, measurements of the resistance were carried out for ozone concentrations less than 120 ppb during an exposure time of 3 minutes at 260 °C. Fig. 6(a–c) shows the dynamic change in resistance during repetitive cycles of alternating exposure to ozone and dry air.

We can observe that the resistance of all films increases during exposure to O_3_ and decreases when dry air is reintroduced into the test chamber. This behavior is the result of adsorption–desorption phenomenon of an oxidizing gas on the surface of an n-type semiconductor.^36^ We also note that the baseline is stable, and all samples have good repeatability indicating the material's potential to detect O_3_ gas. We suggest that the detection mechanism can be explained by dissociative adsorption alone for triatomic gas. Firstly, when ozone is added to the atmospheric gas, O_3_ molecules adsorb on the surface sites of the sensitive layer according to the following equation:

9 O_3_ + e^−^ → O_2_ + O^−^

The trapping of electrons by ozone leads to a decrease of free electron concentration and as a consequence, resistance increases.

In the other side, during the introduction of dry air, O^−^ is desorbed from the surface of the material into the gas phase in the form of ozone molecules as shown in the following equation:

10 O^−^ + O_2_ → O_3_ + e^−^

#### 3.2.2. Ozone sensing performances

##### 3.2.2.1 Response

In order to monitor the effect of the working temperature on the ozone sensor response, the films were heated to five temperatures ranging from 120 °C to 260 °C.

**Fig. 7:**
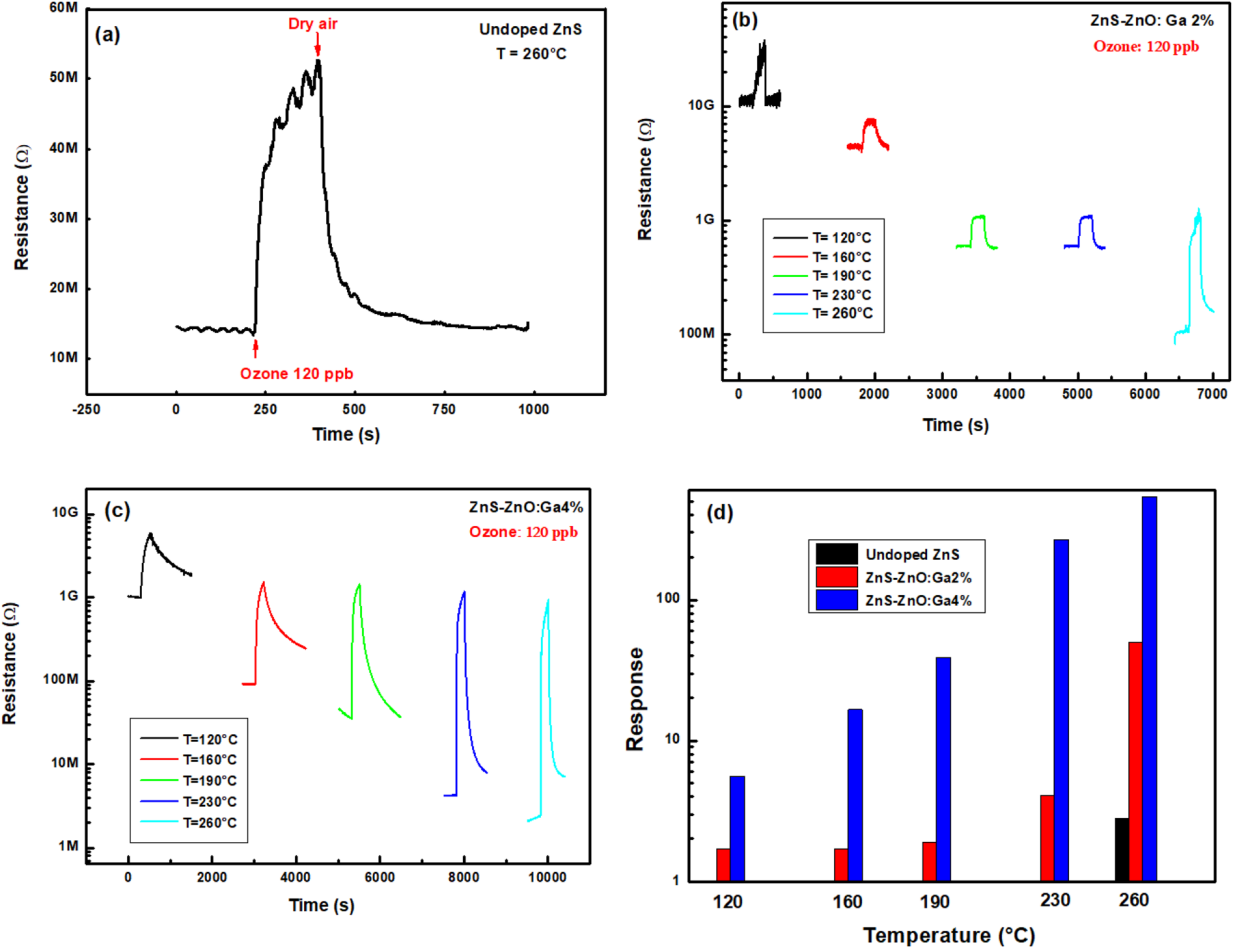
Dynamic change of sample resistance to 120 ppb O_3_ at five working temperatures, (a) undoped, (b) ZnS–ZnO:Ga2%, (c) ZnS–ZnO:Ga4% thin films and (d) response *versus* temperature to 120 ppb ozone.

Fig. 7a illustrates the resistance change of undoped ZnS sample over time for 120 ppb ozone at 260 °C. Below this temperature, the material exhibits high resistance and no signal can be recorded. It now states that the resistance is very high below a certain temperature and it cannot be measured by the Keithley nanoammeter. Fig. 7b and c illustrate the dynamic change in sensor resistance for 120 ppb of ozone at five operating temperatures. We can note that the resistance base line diminishes by rising operating temperature, confirming the semiconductor behavior of the material. Fig. 7d shows that the response increases from 1.7 to 50 for ZnS–ZnO doped Ga2% and from 5.6 to 540 for ZnS–ZnO doped Ga4% by increasing the operating temperature from 120 to 260 °C. While, the undoped layer shows a weak response of 2.8 at an operating temperature of 260 °C. In fact, we think that as the temperature increases, the electron density of the ZnS–ZnO composite increases resulting of the electrons emission from donor levels matching sulfur vacancies, oxygen vacancies and interstitial Zn to the conduction band. This increase in the concentration of free electrons promotes the chemisorption of O_3_ molecules according to eqn (9). In addition, gallium (Ga) is located in group 13, so it therefore has 3 valence electrons, while the zinc has two only. Most importantly, the higher the doping level gets, the higher of the electron density in the ZnS–ZnO composite which further improves the ozone adsorption.^37^ As a result, the sensing response and layer conductivity can be enhanced by Ga doping. ZnS–ZnO:Ga4% is our best candidate to detect ppb level ozone gas.

##### 3.2.2.2 Sensitivity

The experiment was conducted with four different ozone concentrations: 30, 65, 100, and 120 ppb. The sensors were operated at working temperature of 260 °C.

**Fig. 8:**
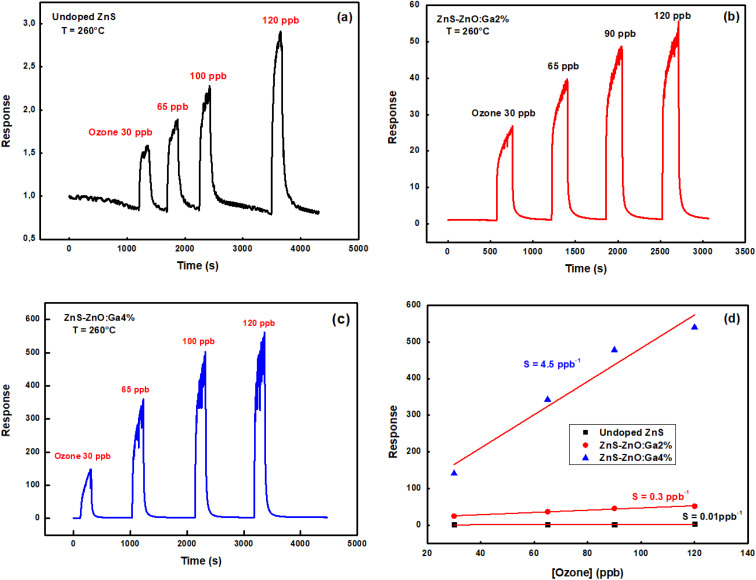
Dynamic response of samples at a working temperature of 260 °C for four concentrations of ozone, (a) undoped, (b) ZnS–ZnO:Ga2%, (c) ZnS–ZnO:Ga4% thin films and (d) response *versus* ozone concentration.

Fig. 8(a–c) show the dynamic response of the sensors to the different ozone concentrations. These results indicate that there is a proportionality between the response of the sensors and the concentration of ozone. We observed that the response of the undoped layer increased from 1.5 to 2.8 by increasing the ozone concentration from 30 ppb to 120 ppb. For ZnS–ZnO:Ga2%, the response increased from 28 to 55. The best response was obtained with the ZnS–ZnO:Ga4% sample, which showed an increase in response from 140 to 540 by increasing the ozone concentration in the same range. Fig. 8d depicts the evolution of the sensor response as a function of ozone concentration. The response is linearly proportional to O_3_ concentration. The sensitivity of the sensor is calculated from the slope of the fitted line and is equal to 0.01 ppb^−1^, 0.3 ppb^−1^, and 4.2 ppb^−1^ corresponding to undoped ZnS, ZnS–ZnO:Ga2%, and ZnS–ZnO:Ga4%, respectively. Consequently, gallium doping improves both sensitivity and response and can be suitable for gas applications.

##### 3.2.2.3 Rapidity

The sensor's performance can be evaluated in terms of its response and recovery times, which are important indicators of its rapidity. The response time is defined as the time necessary for the sensor to achieve 90% of the response reaching a saturate state, and the recovery time represents the time to return to 10% of the baseline after exposure to ozone.

**Fig. 9:**
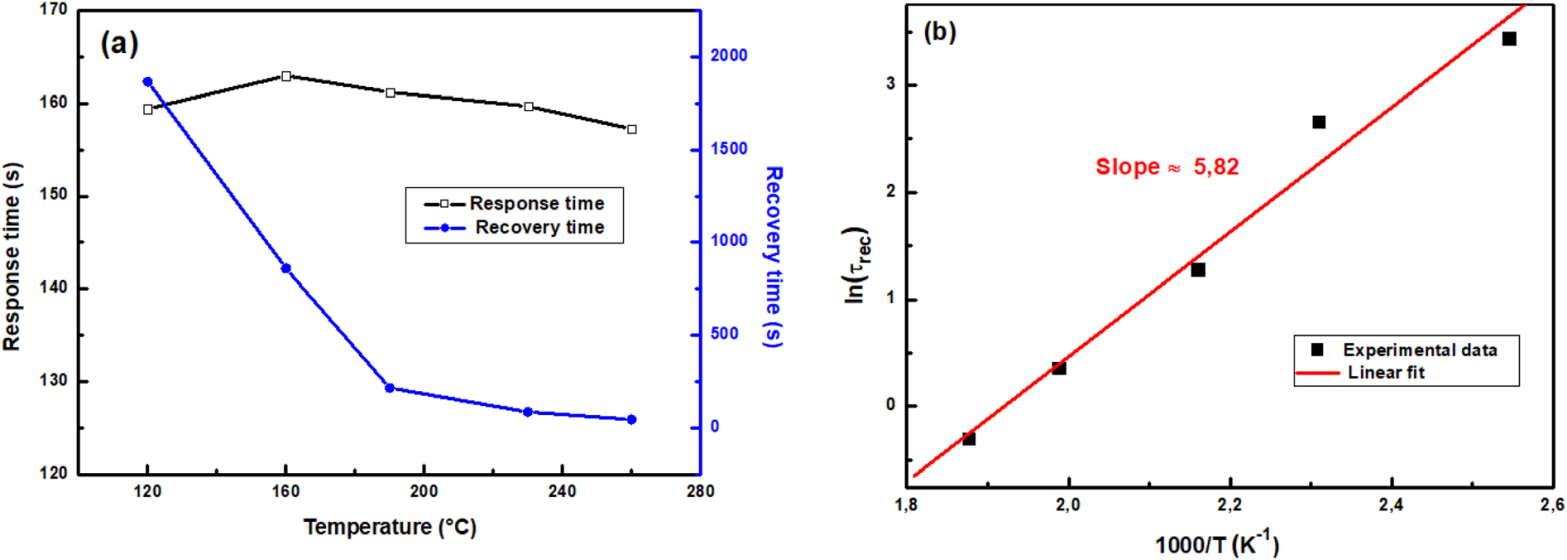
(a) Response time and recovery time of ZnS:Ga4% sensor upon exposure to 120 ppb O_3_ depending on the working temperature. (b) Arrhenius plots of recovery process.

Fig. 9a illustrates the evolution of response and recovery times *versus* working temperature of a ZnS–ZnO:Ga4% sensor exposed to 120 ppb of ozone. It can be noted that the response time is in the order of 160 seconds and the recovery time decreases from 1860 s to 45 s by rising the operating temperature from 120 °C to 260 °C. In fact, when energy is supplied to a system, the temperature rises, which can accelerate the chemical reactions controlling gas adsorption and desorption at the semiconductor surface. With this, the activation energy for the recovery process can be calculated from the dependence of recovery time as a function of temperature, using the well-known Arrhenius equation:11
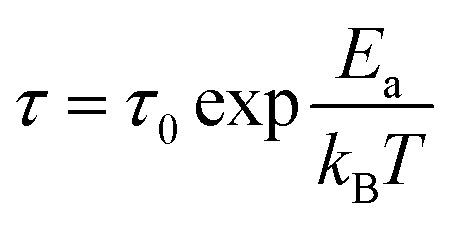
where *E*_a_ is the activation energy required by the sensor for the recovery process for ozone, *k*_B_ is the Boltzmann constant and *T* is the absolute operating temperature of the sensor.

Fig. 9b reveals that ln(*τ*_rec_) varies linearly with 1000/*T*, indicating that the estimated value of the activation energy for the recovery process is 0.5 eV, which is higher than the thermal energy at room temperature (*E*_th_ = 0.025 eV). This result suggests that ozone desorption process is difficult and recovery time is high at low temperature.

##### 3.2.2.4 Selectivity

**Fig. 10:**
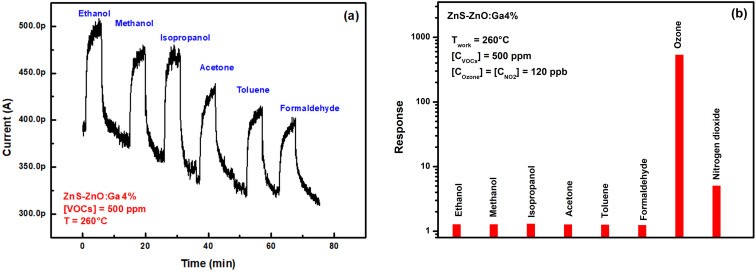
(a) Dynamic change of current to 500 ppm VOCs at 260 °C for ZnS–ZnO:Ga4% sensor, (b) selectivity of ZnS–ZnO:Ga4% sensor.

The selectivity of a sensor refers to its ability to specifically respond to a particular gas in the presence of other gases. To investigate the selectivity of the ZnS–ZnO:Ga4% sensor, the last was exposed to various volatile organic compounds (VOCs) at 500 ppm concentration and nitrogen dioxide (NO_2_) at 120 ppb at a working temperature of 260 °C. Fig. 10a displays the current change *versus* time as the sensor undergoes alternating cycles of exposure to VOCs and dry air. The results of obtained response are illustrated by the histogram in Fig. 10b. We have also reported the response of the same sample for an ozone and a nitrogen dioxide concentration of 120 ppb. The results clearly demonstrate the high selectivity of the ZnS–ZnO:Ga4% sensor towards ozone in presence of VOCs and nitrogen dioxide.

A comparative analysis of the sensing properties of the developed ZnS–ZnO:Ga4% sensor with previously published ozone sensors is presented in Table 2.^38–43^ The results highlight that ZnS–ZnO:Ga4% sensor outperforms other ozone sensors in terms of detection sensitivity. Specifically, it shows promising potential for detecting ozone gas.

**Table 2:** Sensing properties of the developed ZnS–ZnO:Ga4% sensor compared with previously published sensors

Materials	*T* (°C)	LOD (ppb)	Response	*τ*_resp_/*τ*_rec_ (s)	Year	Ref.
β-In_2_S_3_	160	40	1.5	147/414	2022	7
In_2_O_3_	70	200	5	—/—	2021	38
ZnO	250	60	3	9/300	2015	39
CuWO_3_	R.T.	90	10	—/—	2018	40
rGo/ZnO	R.T.	300	1.97	—/—	2018	41
Carbon nanotubes	300	80	1.3	—/—	2019	42
rGo–ZnO	R.T.	100	49.9	—/—	2021	43
ZnS–ZnO:Ga4%	120	120	5	160/1860	2023	This work
ZnS–ZnO:Ga4%	260	30	150	160/45	2023	This work

## 4 Conclusion

In this work, undoped and Ga-doped ZnS–ZnO thin films were deposited on SiO_2_/Si substrates with inter-digitated platinum electrodes by spray pyrolysis technique at a temperature of 350 °C. The effect of Ga doping on structural, morphological, optical and ozone gas sensing properties has been reported. The findings indicate the amorphous structure for the undoped films while with Ga doping the films present two hexagonal phases: ZnS and ZnO. Doping improves considerably crystallinity and the average crystallite size was evaluated to be 18 nm. In terms of surface morphology, the undoped sample was homogenous and smooth, but with doping, it became porous and granular. Moreover, the optical transmission was high in the visible region and reaches 90% in value. The gap energy decreased from 3.84 eV to 3.74 eV by doping. Finally, the ozone detection performances were evaluated. The best properties were found with ZnS–ZnO:Ga4% sensor. At 260 °C working temperature, the response to 30 ppb ozone is 150, the sensitivity is 4.5 ppb^−1^, the response recovery times were found to be 160 s and 45 s respectively. This study suggests that the gallium-doped ZnS–ZnO composite deposited by spray pyrolysis pathway has the potential for highly sensitive and ppb level ozone detection. Nevertheless, these results are encouraging for deepening the study of sensing mechanism to improve performances of ZnS–ZnO:Ga promising sensor.

## Ethical statement

The authors have no potential conflict of interest to disclose.

## Data availability

The datasets generated during and/or analyzed during the current study are available from corresponding author on reasonable request.

## Author contributions

Conceptualization: N. Bouguila, R. Souissi, M. Bendahan. Acquisition of data: T. Laribi, S. Bernardini, R. Souissi. Formal analysis: T. Laribi, R. Souissi, N. Bouguila, S. Bernardini, M. Bendahan, S. Alaya. Methodology: T. Laribi, R. Souissi, S. Bernardini. Supervision: N. Bouguila, R. Souissi. Validation: T. Laribi, R. Souissi, S. Bernardini, M. Bendahan, N. Bouguila, S. Alaya. Writing original draft: T. Laribi, R. Souissi, N. Bouguila. Writing review & editing: M. Bendahan, S. Bernardini, S. Alaya.

## Conflicts of interest

There are no conflicts to declare.

## Supplementary Material
